# Experimental Study on the Influence of Different Factors on the Mechanical Properties of a Soil–Rock Mixture Solidified by Micro-Organisms

**DOI:** 10.3390/ma15207394

**Published:** 2022-10-21

**Authors:** Yongshuai Sun, Ya Tuo, Jianguo Lv, Guihe Wang

**Affiliations:** 1 College of Water Resources & Civil Engineering, China Agricultural University, Beijing 100083, China; 2 School of Engineering and Technology, China University of Geosciences, Beijing 100083, China

**Keywords:** soil–rock mixture, MICP, triaxial compression test, deviatoric stress, shear strain, SEM, XRD

## Abstract

In this paper, we focus on the application of mechanical properties in a soil–rock mixture modified by microbial mineralization under the influence of different factors, including pH value, cementing solution concentration, and cementing time. Cementing fluids and samples with different pH values, calcium ion concentrations, and mineralization cementation were prepared. The process of urea hydrolysis MICP under different factors was studied. A solidified soil–rock mixture sample under triaxial compression was measured. Then, combined with scanning test methods, such as SEM and XRD, the influence of different factors on the mechanical strength and failure mode of the soil–rock mixture structure was analyzed from a microscopic point of view. The results show that a low concentration of cementing solution with a high concentration of bacteria liquid generated the highest calcium carbonate content and the strongest cementing ability. When the pH value of the cementation solution is six, the cementation effect between the pores is the best, and the deviatoric stress is stronger. When wet-curing samples, short or long curing time will adversely affect the strength of soil–rock mixture samples, the strongest curing and cementing ability is 5 days. The microscopic results show that the microbial mineralization technology fills the pores between the particles, and the interaction force between particles is enhanced to enhance the strength of the soil–rock mixture.

## 1. Introduction

The soil–rock mixture in our study was composed of a mixture of soil and rock. It is typical of that widely distributed in nature, with obvious differences in particle size and material composition between the rock and the soil [1,2,3]. Because of the heterogeneity inthe mixture, the slope formed by it has certain instability, which is particularly prominent in geotechnical engineering problems. With the development of various large-scale projects, the problem of slope instability caused by natural stress and human construction is becoming more and more serious. Taking appropriate measures to reinforce the soil–rock mixture slope is of great significance to improve its stability and prevent disasters. Most traditional construction methods use chemical reagents to reinforce the soil. However, chemical reagents are difficult to degrade and thus remain in the soil, causing damage to the ecological environment [4]. Therefore, a new soil reinforcement technology is urgently needed to change the modern construction protocols.

In recent years, microbial-induced calcite precipitation (MICP) has been widely used in geotechnical engineering practice and has achieved good engineering results. MICP refers to the process of synthesizing calcium carbonate from metabolites of specific bacteria and substances in the surrounding environment [5,6]. This technology is inexpensive and less polluting, compared with traditional chemical grouting. It infiltrates soil–rock mixture materials easily [7]. At present, scholars in the geotechnical field mainly focus on the application of MICP technology in soil reinforcement and improvement research. Current research of MICP technology focuses mainly on grouting reinforcement, that is, the relationship between grouting speed, grouting concentration, grouting method, and settlement results. Researchers use laboratory tests to explore the mechanism of action on the strength and stiffness of the soil [8,9,10,11,12]. Tobler [13] compared and studied different grouting methods and found that the parallel grouting method leads to an uneven distribution of calcite. When the bacterial and cementing fluids are injected by the distribution grouting method, a cement sand column with relatively uniform calcite distribution is obtained. Zamani [14] studied the liquefaction resistance of a sand foundation reinforced by microbial grouting through model tests and found that the reinforcement technology based on MICP grouting showed stronger resistance to liquefaction in moderate earthquakes than the traditional reinforcement method of a crushed stone column. Tian [15] studied the influence of different cementation solution injection rates on the physical and mechanical properties and microstructure of the samples based on the aeolian sand MICP technology. Zhang [16] studied the effects of culture medium concentration, substrate concentration, and bacterial inoculation on the deposition rate of calcium carbon at the degree of influence from large to small in substrate concentration, medium concentration, and bacterial inoculation. Duo [17] studied the solidification characteristics of five kinds of cementation solution with different concentrations on sandstone. The analysis showed that with the increase of the cementation solution concentration, as the content of calcium carbonate increased, the unconfined compressive strength increased. After research, scholars used MICP technology to solidify a variety of types of soil. These include sand [18,19,20], silt [21,22], sandy clayey purple soil [23], loess [24,25], expansive soil [26,27], and mucky soil [28,29]. The unconfined compressive strength and cohesion of soil were significantly improved. Xiao [30] used MICP technology combined with the blowing and filling process to solidify coral sand. The stress–strain characteristics of the solidified body were analyzed by a triaxial test, and the results showed that the strength of the cured coral sand reached more than 10 MPa, and the penetration resistance was greatly improved. Xiaohua [31] compared the freeze–thaw resistance of sandstone with different particle sizes before and after MICP treatment and evaluated the improvement effect of MICP technology. Sun [32] analyzed the effect of different temperature conditions on the effect of sand solidification based on sand solidification experiments. It was found that the addition of urea under low temperature acclimation conditions can significantly improve the curing effect. Zhao [33] used MICP technology to conduct sand plugging experiments and found that the microbial mineralization technology can form an effective plug. The results showed that the permeability of sand was greatly reduced. Tan [34] carried out an MICP technology anti-seepage field test. The results showed that the technology can quickly reduce the permeability coefficient of the cohesive soil embankment section. Lu [35] studied the effect of MICP on dam reinforcement through model tests. It was found that MICP technology can effectively improve the erosion resistance of dams. MICP technology has problems, such as an insufficient calcium carbonate crystallization conversion rate and insufficient soil strength. Therefore, scholars proposed that based on MICP, urea [36], poly-Lys [37], egg white [38,39], activated magnesium oxide [40], and fiber [41,42,43] be added, respectively, to treat the soil, the effects of additives with different contents were discussed, and it was noted that the complementary advantages of the two could effectively improve the curing effect of MICP.

Combined with the relevant paper on MICP, current research focuses on the improvement of soil by MICP, and its solidification effect is often restricted and affected by many factors. However, the research on microbial mineralization of the soil–rock mixture is relatively lacking. Based on this, this paper takes the soil–rock mixture as the research object, and the mechanical performance and application prospect of the soil–rock mixture improved by microbial mineralization under the influence of different factors are emphasized. By preparing cementation solutions and samples with different pH values, calcium ion concentrations, and mineralization cementation time, the process of urea hydrolysis MICP under different factors was experimentally studied. The stress–strain relationship of the solidified soil–rock mixture sample under the triaxial compression test was determined, and the change of curing effect under the influence of different factors was analyzed. Then, combined with scanning test methods such as SEM and XRD, the change in the soil–rock mixture was observed from a microscopic perspective. The influence of different factors on the mechanical strength and failure mode of the soil–rock mixture structure was analyzed.

## 2. Microbial Improvement Test of Soil–Rock Mixture

### 2.1. Soil–Rock Mixture

This test selected silt and rock as the object of microbial grouting reinforcement. The soil samples were screened by a multi-functional vibrating screen. The basic physical parameters of silt are shown in Table 1. Small pebbles with a particle size of 0.2–0.4 cm was selected as the rocks in the soil–rock mixture. We use a soil–rock mixture with a rock content of 50% to prepare the sample material as the experimental carrier of the factors affecting cementation in this study.

### 2.2. Bacterial Liquid

**Figure 1:**
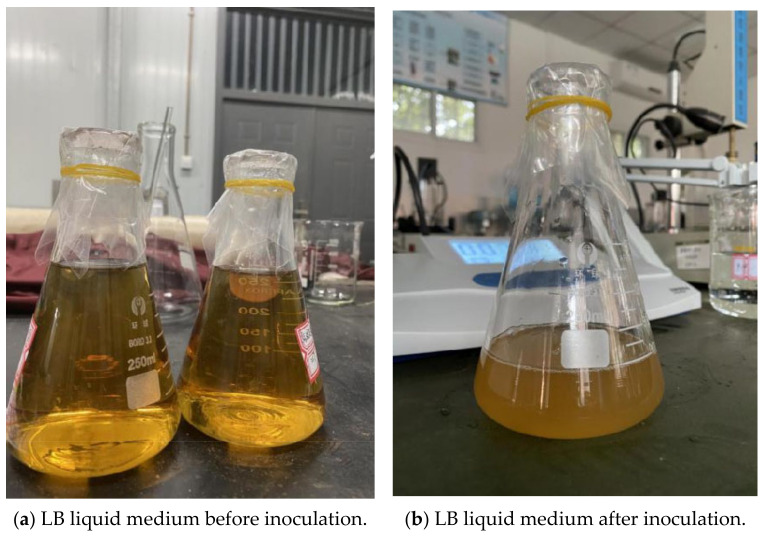
LB liquid medium before and after inoculation.

In this experiment, Sporosarcina pasteurii was used as the test bacterium. After secondary activation and expansion, the experimental bacteria were stored at 4 °C for cryopreservation. The medium used in the experiment was Luria–Bertani (LB) broth liquid medium. The ingredients are listed in Table 2. The prepared culture medium was placed in a conical flask, and the bottle mouth was sealed with parafilm. We sterilized the culture medium, solution, and experimental equipment with high temperature steam at 121 °C for 20 min, then put them on a sterile operating table and waited for cooling. We used a sterile pipette to add 0.5 mL of bacterial liquid into the prepared LB broth liquid medium, and mixed evenly. We placed the conical flask at 30 °C and incubated at 200 rpm in a shaking incubator for 48 h; it was found that the culture medium was obviously turbid, and the bacteria were successfully activated at this time (Figure 1).

Based on the overall analysis of the experiment, this paper uses turbidimetry to determine the number of bacteria. The number of bacteria was obtained by spectrophotometric conversion, which used a spectrophotometer to detect the growth of micro-organisms. To ensure that the bacterial solution had sufficient activity during the test, enzyme activity detection of the cultured bacterial liquid was required. The empirical formula for the relationship between the amount of urea hydrolysis and the change in conductivity was as follows [44]:

Enzyme activity (mM/min) = change in conductivity (mS/cm) ×11.11 (R2 = 0.9988)

During the test, a sterile pipette was used to suck up 1 mL of bacteria solution to be tested. It was mixed with 9 mL of 1 mol/L urea (V bacteria: V urea = 1:9) to measure the urease activity of the bacterial solution by the conductivity method used by Whiffin [44]. We used a conductivity meter to measure the change in the conductivity of the solution within 5 min and multiplied the measured 5-min average conductivity change value by the dilution factor (10 times) to obtain the initial enzymatic activity of the bacterial liquid. This value reflects the ability of microbial urease to hydrolyze urea.

After analysis, the OD600 value (the absorbance value of the bacterial solution at the wavelength of 600 nm was used to characterize the biomass in the bacterial solution.) of the activated and cultured bacterial liquid used in this paper was tested with an ultraviolet spectrophotometer to be 1.25. The original bacterial solution was diluted with physiological saline (0.9% NaCl aqueous solution) to meet the experimental requirements. According to the OD600 value of the bacterial solution and the bacterial concentration, the bacterial concentration converted by the method proposed by Ramachandran [45] was 11.64 × 107 cells/mL.

Cementing liquid is the key to the reaction with bacterial liquid. In this experiment, the Urea–CaCl_2_ mixture was used as the cementing liquid, in which the molar ratio of urea to Ca^2+^ was 1:1.

### 2.3. Test Plan

The test used the fully automatic triaxial instrument of Huakan Technology to carry out the unconsolidated and undrained (UU) test of the cured samples. We set confining pressures of 100 kPa, 200 kPa, and 300 kPa, respectively, and set shear stop strain at 20%. When the strain reaches 20% or the sample is damaged to a certain extent, the instrument automatically stops the test, and calculates the corresponding data. This experiment focused more on the external factors affecting the hydrolysis of bacterial urea. The external factors affecting the hydrolysis of bacterial urea were mainly studied, including the pH value of the cementation solution, the concentration of the cementation solution, and the curing time. By controlling a single variable, the stress–strain characteristics of the solidified soil–rock mixture samples in the triaxial compression test were determined, and the solidification effect was studied and analyzed.

#### 2.3.1. Different pH

We used 0.25 mol/L Urea and 0.25 mol/L CaCl_2_ solution to prepare a 0.25 mol/L cementation solution, and the volume ratio was 1:1. The moisture content of the soil–rock mixture was set to 12%, and the volume ratio of the cementing liquid and bacterial liquid was 1:1. In the prepared cementitious solution, we used potassium acid phthalate or sodium hydroxide to titrate the cementitious solution with four gradients of pH value of 5, 6, 7, and 8, respectively. After testing and calibrating with a pH meter, the cementation solution was poured into the soil–rock mixture, and put in a moisturizing bag for 6 h to ensure that the water molecules of the cementation solution spread evenly in the soil and rock. After exhausting the air, we sealed the tank and let it stand for 6 h, then carried out microbial grouting at room temperature (25 ± 2 ℃). The sample preparation also used a triaxial saturator, and the sample size was 39.1 mm (diameter) × 80 mm (height). Before sample preparation, a certain soil–rock mixture sample was weighed, and a certain quality of cementation solution was added according to the proportion of moisture content. After stirring evenly, we sealed it with a fresh-keeping bag and placed it in a moisturizing tank for 6 h. Before sample preparation, we mixed the 1.25 mol/L bacterial solution with the soil–rock mixture after the tank was stuffed for 6 h.

When using the triaxial saturator to prepare samples, a small amount of Vaseline is smeared in the copper mold to reduce the friction between the sample and the cylinder wall. Samples are prepared according to the quality. Each group prepared 3 soil–rock mixture samples with the same configuration. Each sample was hammered for 30 times, loaded into the mold in 5 layers, and compacted at the same height after being loaded into the mold. The surface of the sample was leveled with a ring knife. Finally, the samples were also demolded and put into a constant temperature and humidity curing box with an ambient temperature of 30 ℃ and an ambient humidity of 70 ± 2% for 5 days.

#### 2.3.2. Different Concentrations

We configure four concentrations of cementation solution (Urea: CaCl_2_ = 1:1): 0.25 mol/L, 0.5 mol/L, 0.75 mol/L, 1 mol/L; the bacterial solution was configured with 1.25 mol/L, the moisture content of the soil–rock mixture was set to 12%, and the volume ratio of cementation solution to bacterial liquid was 1:1. The cementation solution was poured into the soil–rock mixture, placed in a moisturizing bag for 6 h, and then mixed with 1.25 mol/L bacterial liquid. The sample preparation also used a triaxial saturator. After the sample was prepared, the sample was also demolded and placed in a constant temperature and humidity curing box with an ambient temperature of 30 ℃ and an ambient humidity of 70 ± 2% for 5 days, and then the UU test was carried out.

#### 2.3.3. Different Curing Time

To investigate the effect of curing time on MICP-cured samples, four curing experiments were carried out on the cured samples under 4 different time conditions, and 4 times were set: 1 day, 3 days, 5 days, and 7 days. The bacterial concentration of all samples was 1.25 mol/L, the cement concentration was 0.25 mol/L, and urea: CaCl_2_ = 1:1. We set the water content to 12%, and the volume ratio of cement and bacterial liquid to be 1:1. Sample preparation was also performed using a triaxial saturator. After the sample was prepared, the sample was also demolded and placed in a constant temperature and humidity curing box with an ambient temperature of 30 ℃ and an ambient humidity of 70 ± 2% for curing.

#### 2.3.4. Microscopic Observation

Biomineralization is a biological reaction, and the bacteria itself has the characteristics of small size, large number, and rapid response. When micro-organisms act on the soil–rock mixture, the bacterial liquid distribution acts on the gaps between the particles, and the resulting crystals are small in size and have strong dispersibility. Microscopic observation of the soil–rock mixture is required using scanning electron microscopy (SEM) and X-ray diffraction phase analysis (XRD). The energy spectrum analysis and phase analysis of the samples were carried out to explore the material structure, morphology, content, distribution, and other laws of microbial mineralization. The SEM was a Zeiss ZEISS Gemini SEM 300 electron microscope; the maximum magnification can be 1 million times; and the extremely tiny material structure can be observed. XRD is a technique for analyzing the structure of materials by utilizing the diffraction effect of X-rays in crystalline materials.

A small amount of soil–rock mixed broken block adhered to the conductive adhesive, and the ZEISS Gemini SEM 300 scanning electron microscope was used to record the acceleration voltage of 3 kV. The sample was scanned and the morphological characteristics of the soil–rock cementation were photographed. The broken block in the center of the soil–rock column was taken, the sample was ground to below 200 mesh, sieved, and then the chemical composition of the crystal in the sample was analyzed by tableting method.

## 3. Experimental Results and Microscopic Observation Analysis

The subsequent figure shows the results of the uniaxial unconfined compressive strength test of the microbial cemented soil–rock mixture under different working conditions. The compression tests of different pH values, cement concentration, and curing time in the diagram were carried out, and three samples were made for each group. In order to reduce the data error in the experiment and observe the law more intuitively, the average value of the experimental data with the most obvious failure mode under the same conditions was taken as the basis and data support for the following charts.

### 3.1. Influence of the pH Value of the Cementation Solution on the Cementation Effect of the Soil–Rock Mixture

**Figure 2:**
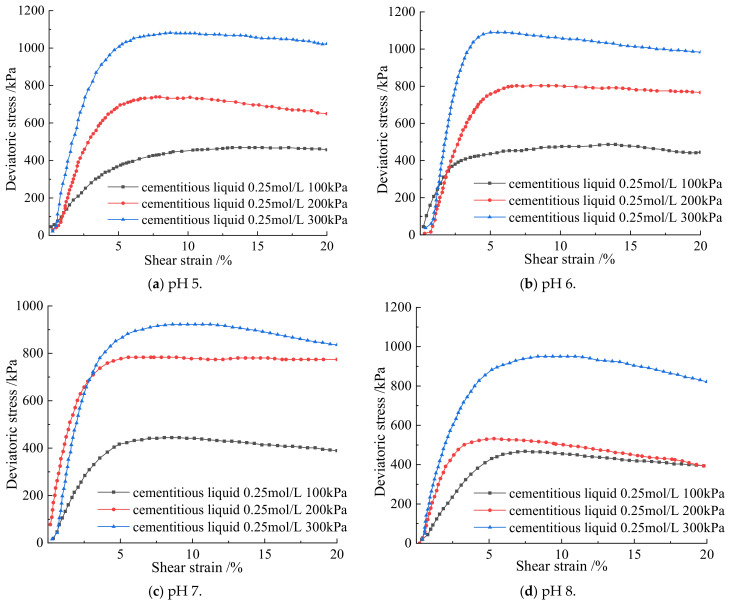
Relationship between shear strain and deviatoric stress of soil–rock mixture.

**Figure 3:**
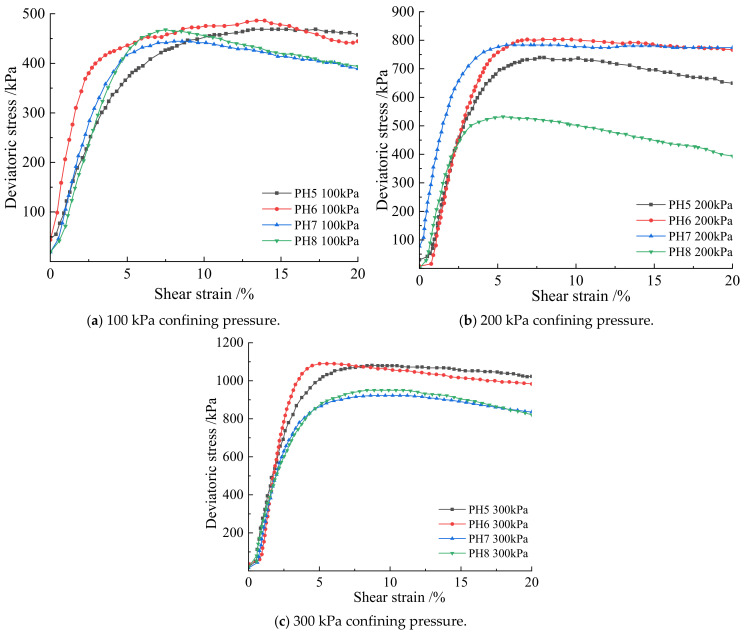
Deviatoric stress at different pH values under different confining pressures.

From Figure 2, when the pH values are 5, 6, 7, and 8, the shear strain and deviatoric stress of the soil–rock mixture are positively correlated under the three relationships of 100 kPa, 200 kPa, and 300 kPa, and the growth rate of deviatoric stress decreases with the increase of shear strain and finally reaches a steady state. It can be seen from Figure 3 and Table 3 that under the same confining pressure, the soil sample fails at 3–5% strain. Under the confining pressure of 100 kPa, the deviatoric stresses of pH 5–8 are 464.6 kPa, 487.3 kPa, 445.6 kPa, and 470.8 kPa, respectively. Among them, the deviatoric stress of pH 6 is the largest, which is 487.3 kPa; under the confining pressure of 200 kPa, the deviatoric stress of pH 5–8 is 740.6 kPa, 806.2 kPa, 787.9 kPa, 536 kPa, and 200 kPa, respectively. The deviatoric stress of pH6 is the largest, which is 806.2 kPa; under the confining pressure of 300 kPa, the deviatoric stresses of pH 5–8 are 1082.5 kPa, 1097 kPa, 926.9 kPa, and 955.2 kPa, respectively. Under the confining pressure of 300 kPa, the deviatoric stress of pH 6 is the largest, which is 1097 kPa. Under the same confining pressure, the peaks of deviatoric stress all appear in the soil–rock mixture sample at pH 6. It shows that when the pH value of the cementation solution is six, the cementation effect between the pores is the best, and the deviatoric stress that can be carried is stronger. Therefore, with the increase of pH value, the ultimate deviatoric stress of the sample first increases and then decreases.

**Figure 4:**
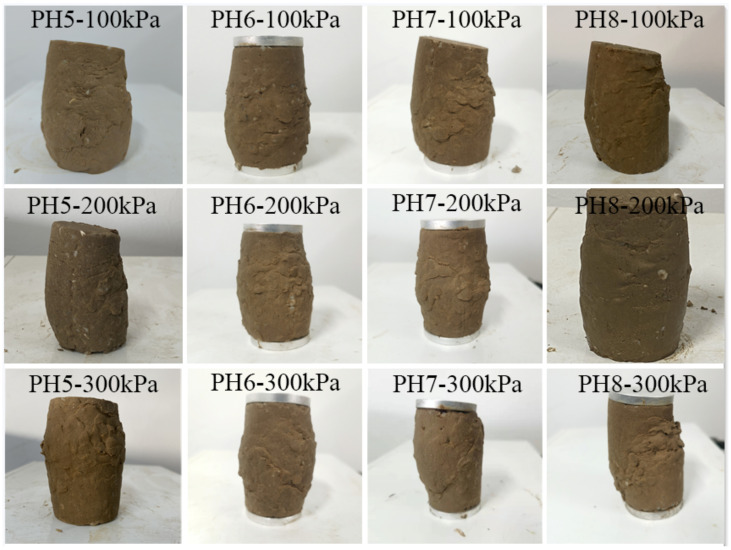
Samples after the triaxial shear test.

Observing Figure 4, after the triaxial shear test of the soil–rock mixture, bulging and shear planes appeared. The samples with pH 7 and pH 8 showed obvious shear planes at 100 kPa, 200 kPa, and 300 kPa. The middle section of the sample bulges outwards, the diameter becomes larger, and the failure surface is an oblique straight shear plane. It is in the double failure state of fine-grained soil and saturated state, indicating that in the simulated curing environment of 70 ± 2% ambient humidity, under the action of water molecules, the bacteria have been performing urea hydrolysis and mineralization inside the sample column.

### 3.2. Effect of Cementation Solution Concentration on the Cementation Effect of Soil–Rock Mixture

**Figure 5:**
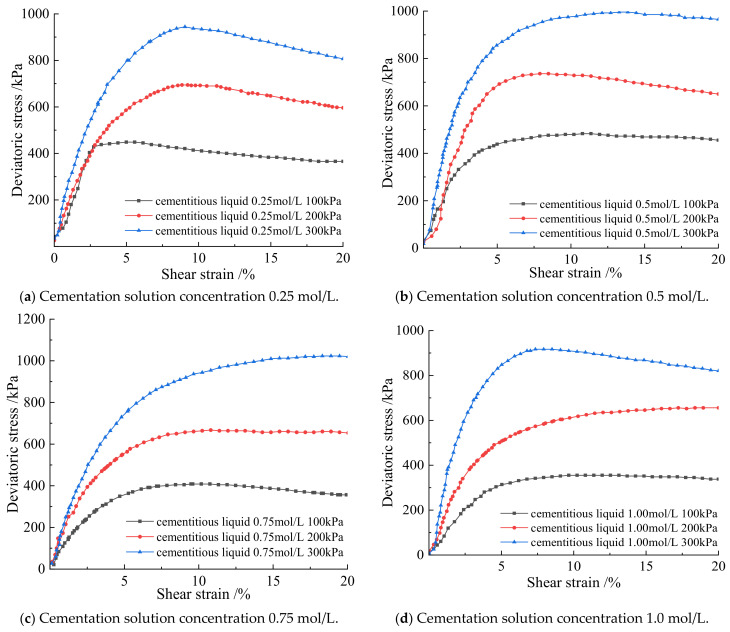
The relationship between shear strain and deviatoric stress of the soil–rock mixture under different cementation solution concentrations.

From Figure 5, when the cementation solution concentration is 0.25 mol/L, 0.5 mol/L, 0.75 mol/L, and 1.0 mol/L, the deviatoric stress of the microbially cemented soil–rock mixture increases with the increase of the shear strain, and the deviatoric stress of the soil–rock mixed column under high confining pressure is higher than that of the soil–rock mixed column under low confining pressure. at the same concentration, the deviatoric stress of the soil–rock mixed column increases with the increase of the confining pressure and tends to be stable gradually. The variation trends of the stress–strain curves of the microbial soil–rock mixture cemented cylinder under the three confining pressures are the same. When the cement concentration is 0.25 mol/L, the three stress–strain curves all increase with the increase of shear strain, an inflection point appears, and an extreme vertex appears, which is the peak of the curve. At this time, it is in the elastic–plastic stage, and the structural strength reaches the maximum. It shows that the combination of 0.25 mol/L cementing liquid and bacterial liquid has achieved initial results in the soil–rock mixture, calcium carbonate crystals are formed by sufficient contact between particles, and the consolidation ability of the soil–rock mixed cylinder is strengthened. When the concentration of the cementing liquid is 0.5 mol/L, in the shear strain of 0–5%, the slope of the stress–strain curve is large, and there is an obvious peak. It shows that in the cement concentration of 0.5 mol/L, the bacterial liquid reacts with the cement quickly, and the maximum strength is reached first. When the cement concentration is 0.75 mol/L, plastic deformation occurs, and the curve has an upward trend as a whole, with no obvious peak, showing a smooth curve. When the cement concentration is 1.0 mol/L, the peaks of the curves under the three confining pressures are lower than those of other concentrations, and the strength is slightly lower than that of other cement concentrations. A low concentration of cementation solution is conducive to microbial mineralization, too high a concentration inhibits urea hydrolysis and crystallization, the pore filling force between particles is low, and shear deformation is more likely to occur under the action of force.

**Figure 6:**
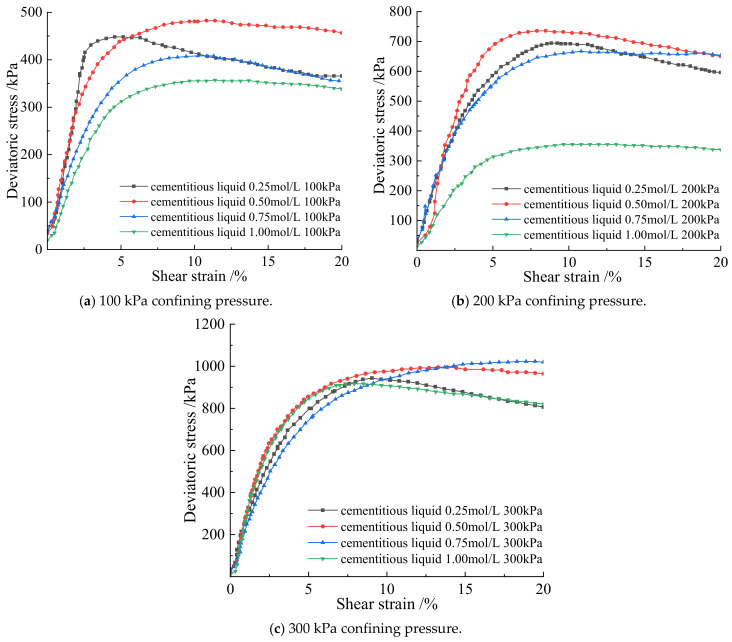
Deviatoric stress at different confining pressures with different cementation solutions.

From the analysis of Figure 6 and Table 3, it can be seen that when the bacterial concentration is constant and the curing time is constant under three different confining pressures, the cementation solution concentration of 0.5 mol/L has the best degree of cementation and the strongest shear resistance. Combined with Figure 5 and Figure 6 and Table 3 under the same confining pressure and 1.25 mol/L bacterial solution under the constant temperature and humidity conservation conditions, the strength of the strength of cementation and mineralization with different concentrations of cementation solution from large to small concentration is 0.5 mol/L, 0.25 mol/L, 0.75 mol/L, and 1.0 mol/L.

**Figure 7:**
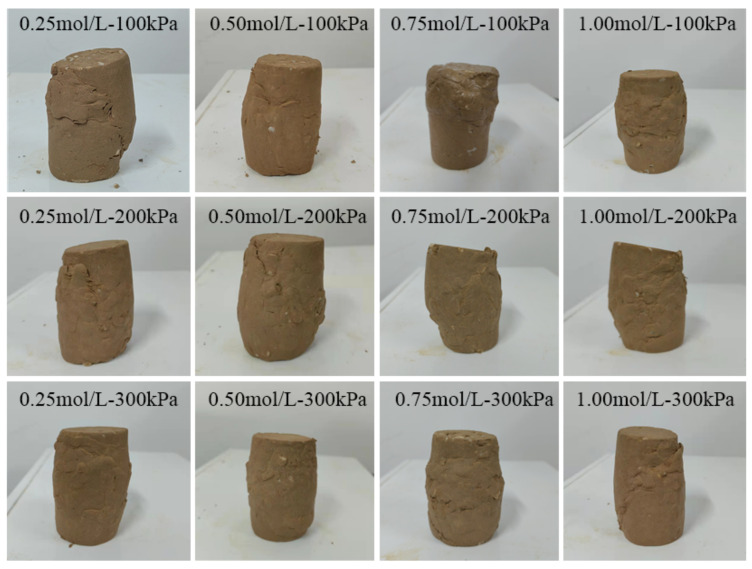
Specimen after the triaxial shear test.

According to Figure 7, when the soil–rock mixture sample cylinder is observed laterally under three different confining pressures when the cement concentration is 0.25 mol/L, the samples all showed a shear plane with a diagonal line from top to bottom. The samples are distorted at the contact surface of the failure surface, there are rocks falling off, the middle of the whole is bulging, and there is an obvious sliding and sliding trend at the shearing part. When the cementation solution concentration is 0.5 mol/L, the middle part of the sample is bulging, there is a shear plane, and the degree of soil–rock slippage is not obvious. It shows that the micro-organisms fully hydrolyze the urea in the sample body, and the soil–rock mixture sample forms a skeleton under the mineralization. Combined with Table 3, the ultimate deviatoric stress of the sample is higher at this time. When the cementation solution concentration is 0.75 mol/L, it is obviously observed that under the confining pressure of 100 kPa, the soil liquefaction phenomenon appears at the top of the sample cylinder, and the surface of the cylinder has obvious shear planes. Under the confining pressure of 200 kPa and 300 kPa, there is obvious shear deformation, and some samples have slip phenomenon. When the cement concentration is 1.0 mol/L, the compression deformation of the sample is serious, the middle of the column bulges, and the shear plane is obvious. Under the confining pressure of 300 kPa, the sample at the broken place has a tendency to slide downward. Longitudinal observation of the cemented cylinder is shown in Figure 7. The shear plane of the specimen under high confining pressure is less damaged than that of the specimen under low confining pressure. In general, the failure degree of the sample cylinder mixed with soil and rock under high confining pressure is lower than that of low confining pressure. The bulge in the middle of the sample under 300 kPa confining pressure is less than that under 100 kPa.

When the sample is compressed under high confining pressure, the pores between the samples are compressed, and the overall volume of the cylinder is greatly compressed. The repulsive force between particles increases, and the overall strength increases, indicating that larger deviatoric stress is required to destroy the cylinder under high confining pressure. The shear strength of the soil–rock cemented mixture under high confining pressure is higher than that of the low confining pressure.

### 3.3. Effect of Curing Time on the Cementation Effect of Soil-Rock Mixture

**Figure 8:**
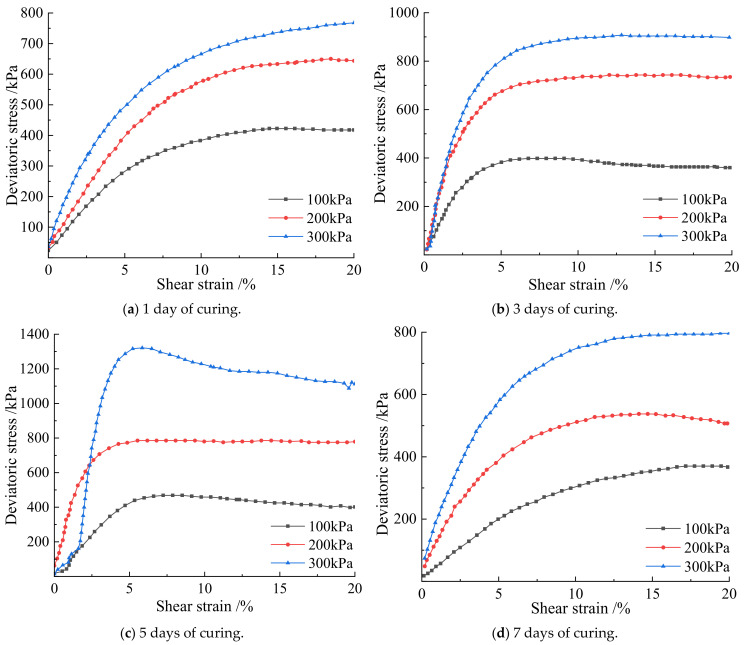
The relationship between shear strain and deviatoric stress of soil–rock mixture under different curing time.

According to Figure 8, the shear strain and partial stress of the earthwork mixture in 1 day, 3 days, 5 days, and 7 days were positively correlated at 100 kPa, 200 kPa, and 300 kPa, and finally reached a steady state of the four different groups of curing times. When the curing time is 1 day, under the three confining pressures, the stress–strain curves all show a weak hardening state, and the rocks are approximately suspended in the fine-grained soil. The soil–rock mixture sample did not form a skeleton, the curing time of the sample was short, and the microbial mineralization and cementation were poor. The micro-organisms have not fully reacted with the calcium ions in the cementation solution, and the ultimate deviatoric stress is low. When the curing time is 3 days, the deviatoric stress under each confining pressure increases with the increase of shear strain, and all showed a “turning point”, indicating that the microbial mineralization and cementation achieved initial results on the third day, and the bacteria liquid and cementation liquid reacted sufficiently. It is converted into calcium carbonate crystals during the contact time, which strengthens the consolidation ability of the soil–rock mixed cylinder; When curing for 5 days, the stress–strain curves under each confining pressure show obvious peaks. This indicates that under the action of microbial mineralization, the originally weak occlusal effect was strengthened between the mixed particles of soil and rock, the rearrangement of soil particles, the resulting calcium carbonate crystals filling the pores between the soil and rock particles, and further reducing the pores. The deviatoric stress reaches the peak strength, which greatly enhances the shear resistance of the soil–rock mixed column. When curing for 7 days, the peak value of the stress–strain curve disappears, and the whole is in a state of plastic deformation. The soil and water saturation capacity of silt is poor, and long-term exposure to a humid environment leads to soil saturation, Under the action of stress, relative displacement occurs between particles, the pores become smaller, the pore water between the particles is squeezed before it can be excreted, the friction force between soil and rock particles gradually decreases to zero, and the strength is lost, resulting in soil liquefaction. The overall deviatoric stress is low.

**Figure 9:**
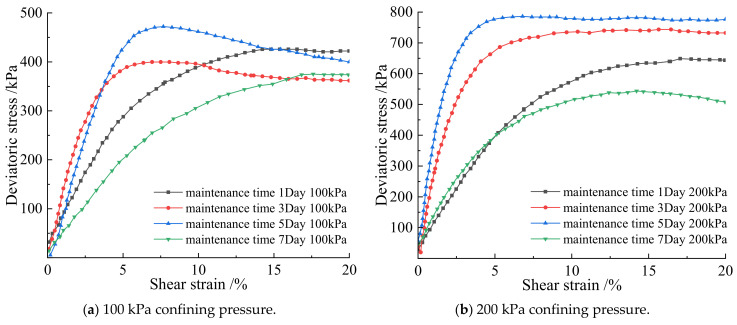

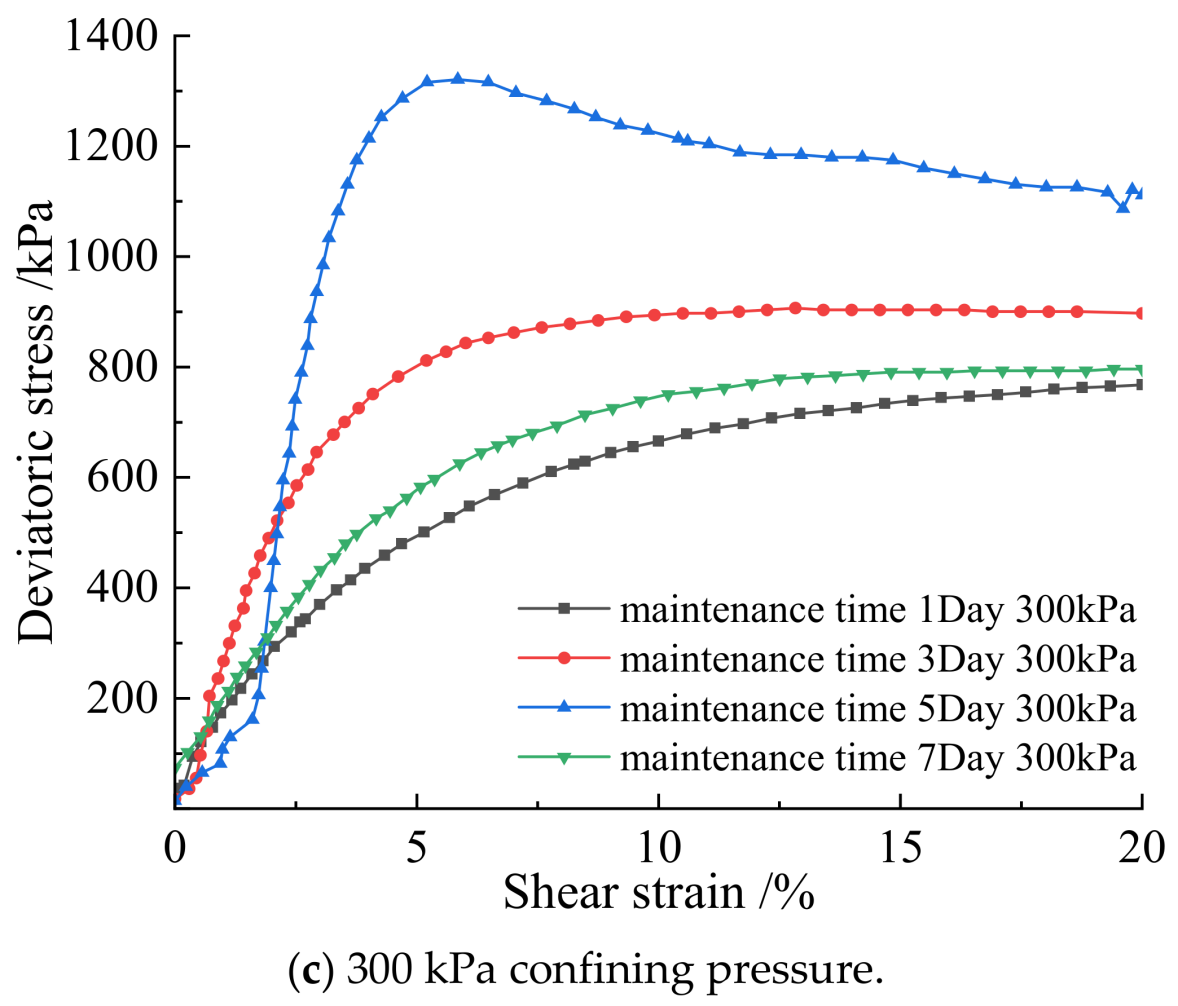
Deviatoric stress at 300 kPa confining pressure for different curing times.

Analyzing Figure 9 and Table 4, under the same confining pressure, obvious soil failure occurs when the strain is 3–8%. Under the confining pressure of 100 kPa, the deviatoric stresses reached by curing time of 1 day, 3 days, 5 days, and 7 days are 399.8 kPa, 425.7 kPa, 470.6 kPa, and 373.5 kPa, respectively, and the curve has obvious peaks. Among them, the deviatoric stress of curing for 5 days under the same conditions is the largest, 470.6 kPa. The deviatoric stresses under the confining pressure of 200 kPa are 649.5 kPa, 745.2 kPa, 788.4 kPa, and 544.2 kPa, respectively. Under the confining pressure of 200 kPa, the deviatoric stress of curing for 5 days is the largest, which is 788.4 kPa. Under the confining pressure of 300 kPa, the deviatoric stresses are 771.4 kPa, 904.2 kPa, 1321.1 kPa, and 800.6 kPa, respectively. Under the confining pressure of 300 kPa, the deviatoric stress is also the largest for 5 days, which is 1321.1 kPa. Comprehensive analysis shows that the soil–rock mixed column cured for 5 days has the best cementation performance, and the cementation ability of microbial mineralization is the strongest. Under the same confining pressure, the peaks of deviatoric stress all appeared in the soil–rock mixture samples cured for 5 days. In the process of simulating the real underground soil environment, water molecules play an important role in the process of microbial mineralization. Water can increase the saturation in the soil–rock mixture sample; it can also provide hydroxide ions necessary for the hydrolysis of bacterial urea in the process of microbial mineralization. It makes the bacteria grow continuously in the soil and rock samples, cementing to form calcium carbonate crystals, which strengthens the strength of the samples. The humidity curing environment full of water molecules is favorable for the cementation of the soil–rock mixture in a short time, and the degree of cementation increases with time. When the curing of the wet environment makes the soil reach saturation, the continuous increase of water molecules will lead to the reduction of the friction between particles, and the soil liquefies, resulting in a decrease in strength. The samples from 1 to 5 days belong to the stage of soil–rock cementation and mineralization optimized by water molecules, and the strength of the samples increases sequentially. After 5 days of curing, the optimum time is reached, and the compressive strength is the largest; when the soil is cured for 7 days, the supersaturation of the soil leads to the liquefaction of the soil, and the strength decreases.

**Figure 10:**
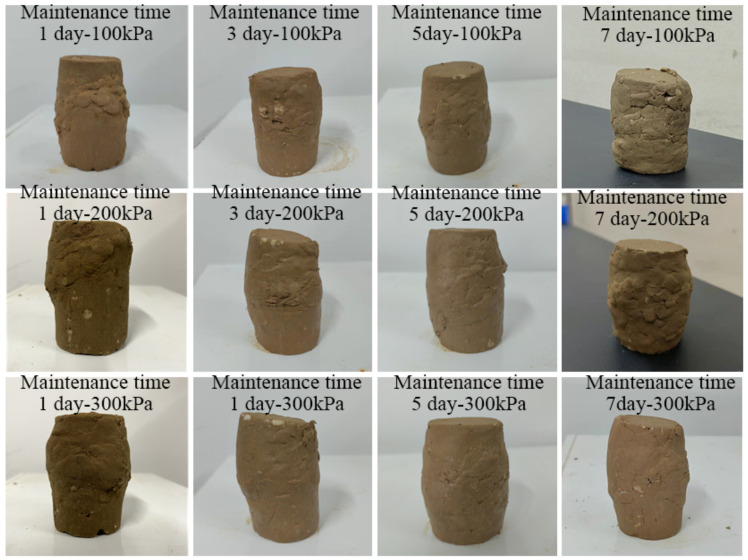
Specimens after triaxial shear test under different curing time.

Observing the soil–rock pillars in Figure 10 and Table 5, after the triaxial shear test, bulging appeared in the middle of the sample, and all produce an oblique diagonal shear surface. The shear failure surface of some samples also has block samples falling off. Through horizontal comparison, it is found that under the same confining pressure and different curing time, the soil–rock mixture samples cured for one day have obvious shear planes. This shows that the bacterial liquid cementation is not sufficient, and a large number of calcium carbonate crystals have not been generated between the soil particles. The cementation strength of the earth rock is poor, and the strength of the earth rock is more dependent on the skeleton structure of the earth rock itself. Therefore, during the compression process of the sample, the content of calcium carbonate cement formed between the pores of the soil and rock particles is less, insufficient to tightly combine silt and rocks. The space between pores is reduced. Without a large amount of calcium carbonate crystal support, the occlusal effect between the mixed particles of soil and rock is small, the supporting force is weak, the ultimate deviatoric stress is low, and there is an obvious shear plane. The bulging dimension in the middle of the sample column after curing for 3 days is significantly smaller than that after curing for 1 day. The shear plane is inclined diagonally from the top to the middle or bottom, and the overall deformation degree is less than that of the sample cured for 1 day. This shows that the micro-organisms begin to mineralize in the voids, bond the silt and rocks, enhance the cohesion, and strengthen the strength of the sample. When cured for 5 days, the overall deformation degree is less than that of the samples cured for 1 and 3 days, the middle of the cylinder is bulging, there are shear surface traces, and there is no obvious damage point on the surface. It shows that the bacterial liquid and the cementing liquid react sufficiently and are effectively converted into calcium carbonate crystals within the contact time. It fills the pores between the particles, binds the silt and rocks, and strengthens the consolidation ability and shear resistance of the soil–rock mixed cylinder. After curing for 7 days, the sample shows obvious damage, the sample collapses, and the shear surface is obvious. It shows that in the process of wet curing, excessive water molecules cause the silt to liquefy, the pore water between the particles is not drained, and is squeezed. The friction between the particles is small, the overall deviatoric stress is low, and the shear strength of the sample is low. In triaxial compression, the shear plane is inclined from top to bottom. It can be observed that shear failure increases the failure path when encountering rocks on the surface of the sample. The new path of destruction unfolded along the shape of the rock, and the depth of the inlay causes part of the rock to fall off, for example, samples cured at 100 kPa for 3 days and 7 days and samples cured at 200 kPa for 7 days. Longitudinal observation shows that the damage degree of the sample cylinder with mixed soil and rock under high confining pressure is generally lower than that of low confining pressure, and the bulge in the middle of the sample under 300 kPa confining pressure is less than that under 100 kPa. It shows that the silt particles undergo great volume compression under high confining pressure. Under high confining pressure, the clay will undergo great volume compression, the pores will shrink, the repulsion between particles will increase, the overall strength is improved, and the body strain is lower than that of the sample body under low confining pressure. If the cylinder is to be destroyed, larger deviatoric stress is required, and the shear strength of the soil–rock cemented mixture under high confining pressure is higher than that of the low confining pressure specimen.

### 3.4. Microscopic Observation of the Effect of Different Factors on the Cementation of Soil–Rock Mixture

#### 3.4.1. Microscopic Observation of the Effect of the pH Value of the Cementation Solution on the Cementation of the Soil–Rock Mixture

The soil–rock mixture samples containing cementation solution pH 6 and pH 8 were selected to observe the microscopic morphology of cementation under different cementation solution PH.

**Figure 11:**
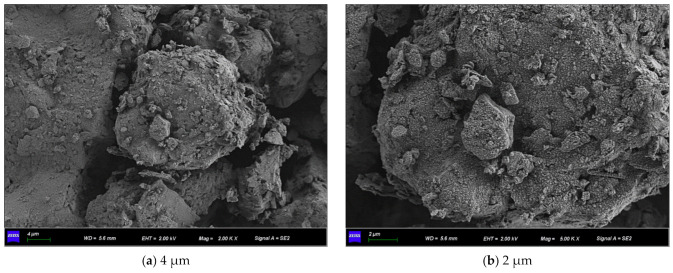
SEM micrographs of cement solution pH 6 under different magnifications.

**Figure 12:**
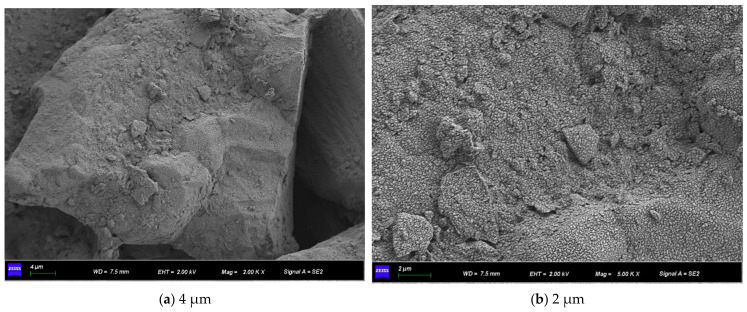
SEM micrographs of cement solution pH 8 under different magnifications.

Figure 11 shows the SEM micrographs observed under three different magnifications of the microbially mineralized soil–rock mixture at pH 6 of the cementitious solution. Figure 12 shows the SEM micrographs observed at three different magnifications for the microbially mineralized soil–rock mixture at pH 8 of the cementation solution. It can be observed that there is a clear formation of calcium carbonate crystals between the particles. Calcium carbonate crystals play a role in bonding the soil and rock and filling the pores between the particles between the soil and rock, strengthening the strength of the sample. From Figure 11 and Figure 12, when the pH value of the cementation solution is 6, calcium carbonate crystals are attached to the rocks in a mass form and bond with the silt on the contact surface of the rocks. When the pH of the cementation solution is 8, the calcium carbonate crystals are flocculent and punctate, and there is obvious cementation between the silt blocks, which is consistent with the mechanical test results in Section 3.1.

**Figure 13:**
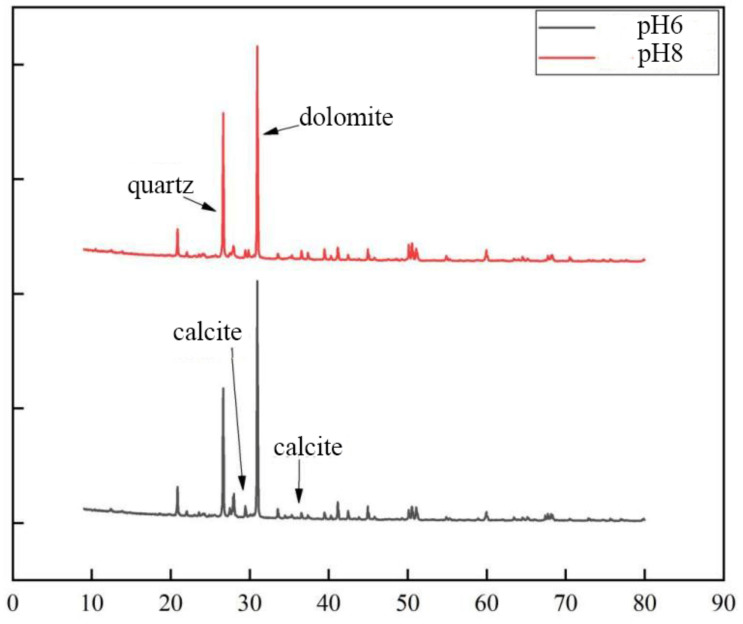
XRD patterns of samples at different pH.

**Figure 14:**
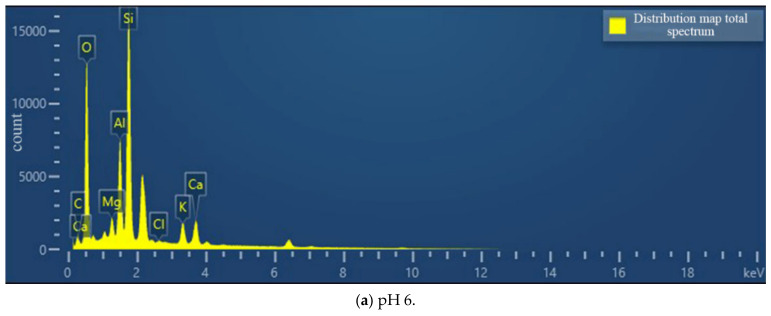

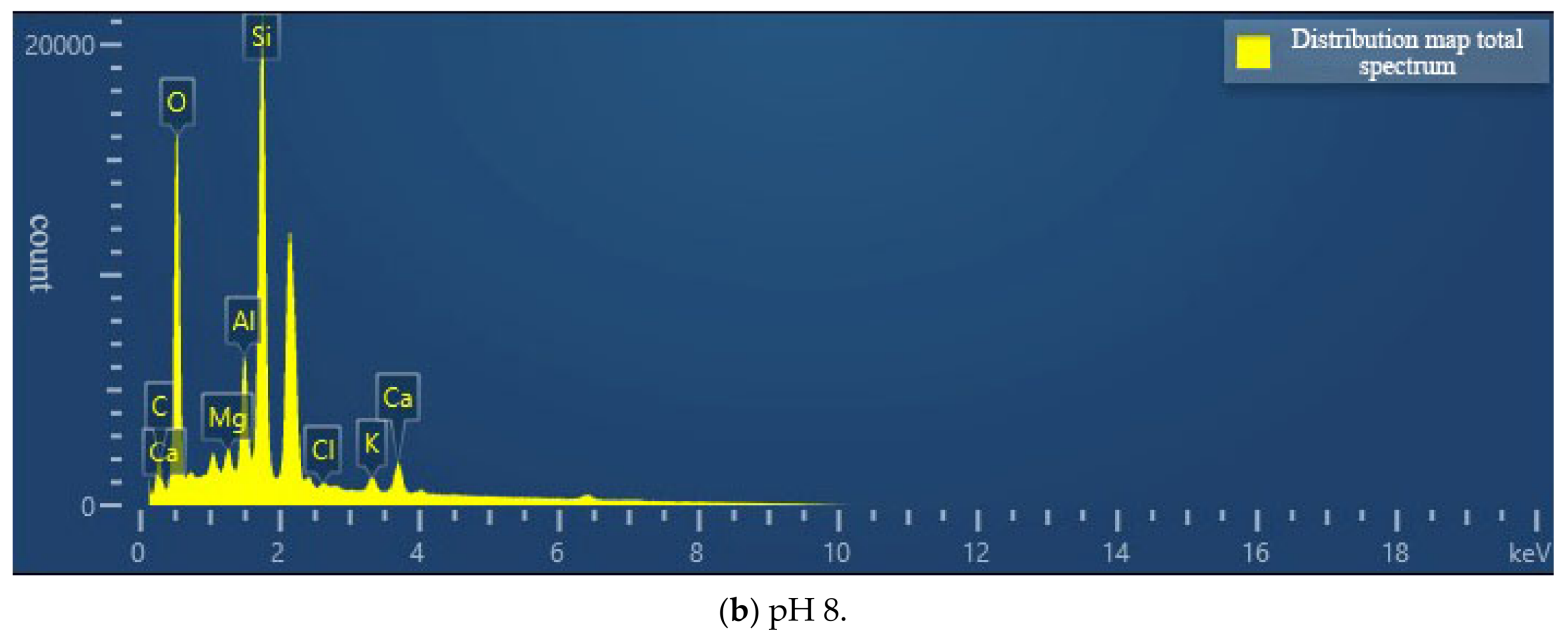
Element distribution map under different cementation pH.

According to Figure 13, the contents of calcite and dolomite produced in the cementing solution at pH 6 are both greater than those in the cementing solution at pH 8. Therefore, the combination of weak acid cement and bacterial liquid may generate more calcium carbonate crystals. Combined with the EDS composition analysis results of cement pH 6 and cementitious solution pH 8 (Figure 14), the percentage of calcium ions in the sample at pH 6 is also higher than that at pH 8, which is consistent with the conclusion of the mechanical test. It shows that in the process of growth and metabolism of bacteria, the surrounding environment is weakly alkalized. An overly alkaline environment may inhibit the growth of micro-organisms. A weak acid is needed to neutralize the soil environment to provide a suitable external growth environment for the growth of bacteria, which is conducive to the growth of micro-organisms. More calcium carbonate crystals are precipitated to enhance the strength of the sample.

#### 3.4.2. Microscopic Observation on the Effect of Cement Concentration on the Cementation of Soil–Rock Mixture

The soil–rock mixture samples containing cementation solution concentrations of 0.5 mol/L and 1.0 mol/L were selected to observe the microscopic morphology of cementation at different concentrations of cementitious solution.

**Figure 15:**
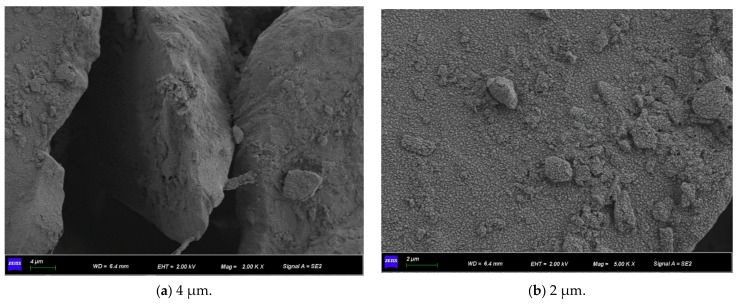
SEM micrographs of 0.5 mol/L cementation solution under different magnifications.

**Figure 16:**
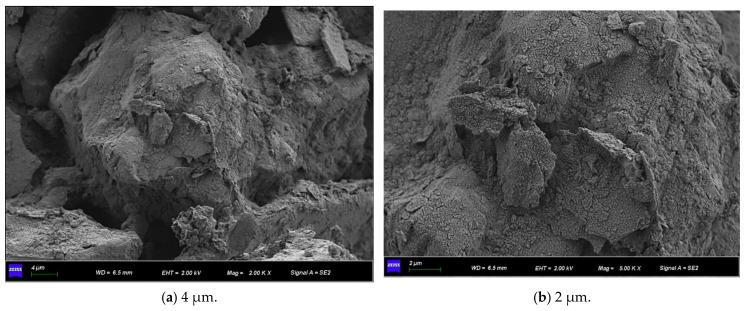
SEM micrographs of 1.0 mol/L cementation solution under different magnifications.

Observing Figure 15 and Figure 16, both of them formed flocculent calcium carbonate crystals on the surface of the sample, the aggregation phenomenon is obvious at the pores of the particles so that the silt particles are aggregated and cemented into agglomerates, and there is an obvious connection effect at the “clumps”. It is proved that the resulting calcium carbonate crystals can strengthen the connection between soil particles when they act on soil particles, fill the pores, make the structure more compact, and strengthen the strength of the soil.

**Figure 17:**
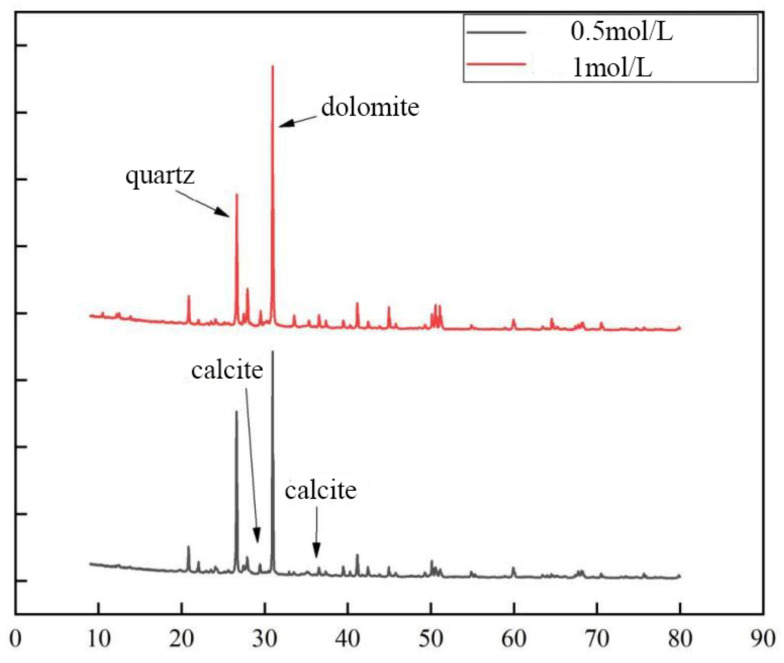
XRD patterns of samples at different concentrations.

**Figure 18:**
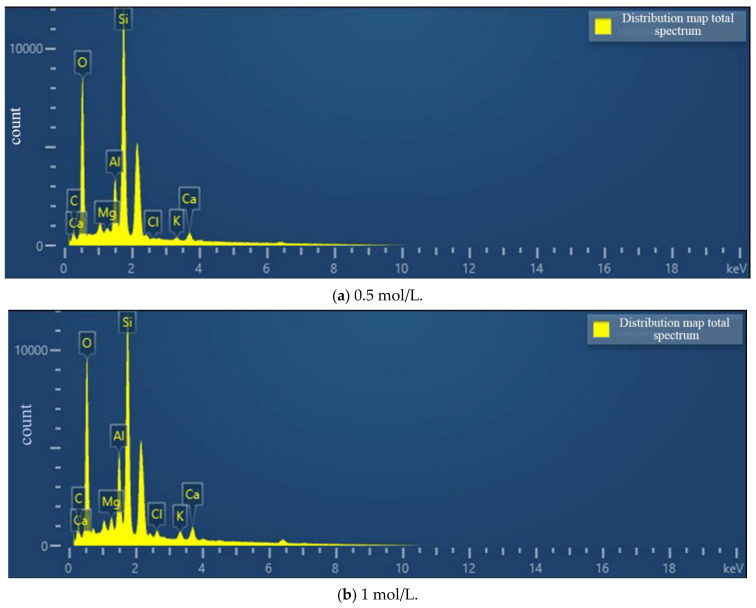
Elemental distribution of different cementitious liquid concentrations.

It can be seen from Figure 17 that the content of crystals generated when the cement concentration is 1.0 mol/L is less than that when the cement concentration is 0.5 mol/L. It shows that the too high concentration of cementitious solution inhibits the formation of calcium carbonate, which is inversely proportional to the calcium ion content of the sample in the SEM (Figure 18). It is further explained that excessive concentrations of cementitious liquid and bacterial liquid cannot fully react, which will cause “excess” calcium ions to exist in the soil in a free manner. This part of calcium ions does not participate in the mineralization of micro-organisms but inhibits the urea hydrolysis of bacteria, resulting in fewer calcium carbonate crystals. This is consistent with the mechanical test in Section 3.2, which further indicates that the high concentration of cementitious liquid is not conducive to the cementation and mineralization of micro-organisms.

#### 3.4.3. Microscopic Observation on the Effect of Different Curing Times on the Cementation of Soil–Rock Mixture

We select soil–rock mixture samples with curing for 5 days and curing for 7 days and observed the microscopic morphology of cementation at different curing times.

**Figure 19:**
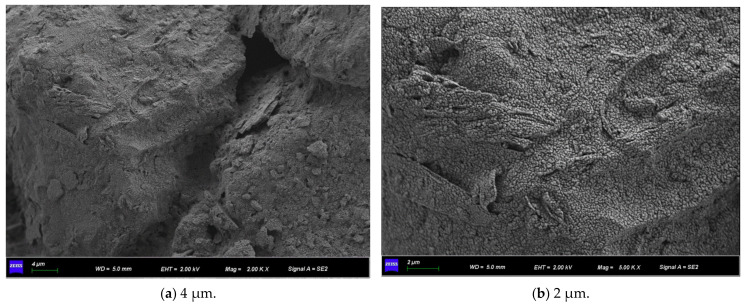
SEM micrographs of curing for 5 days at different magnifications.

**Figure 20:**
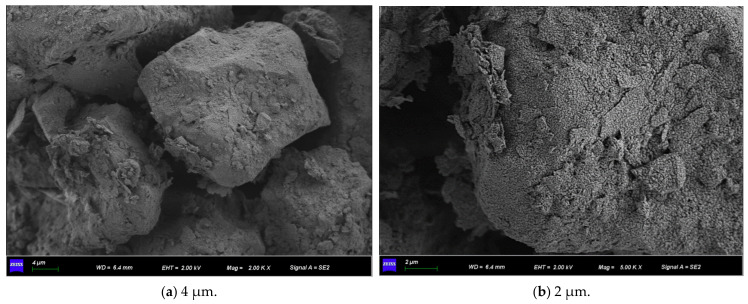
SEM micrographs of curing for 7 days at different magnifications.

Observing Figure 19 and Figure 20, the calcium carbonate crystals after curing for 5 days are in the form of tiny fragments. They gather and adhere to the pore surface of the soil and rock and are “cracking” on the soil surface. Microorganisms are flake-like aggregated crystals on the samples cured for 7 days; the crystals attached to the surface are significantly more than those of the samples cured for 5 days, indicating that more calcium carbonate crystals were formed. Since the whole experiment was in a wet curing environment, combined with the results of mechanical experiments, when curing for 1–5 days, the strength of the sample increases with time, and the overall strength shows an upward trend. At 7 days of curing, the silt has poor saturation capacity. In a humid environment for a long time, the soil will soon reach saturation, the friction between particles will decrease, and soil liquefaction occurs. So, the overall stress is low.

**Figure 21:**
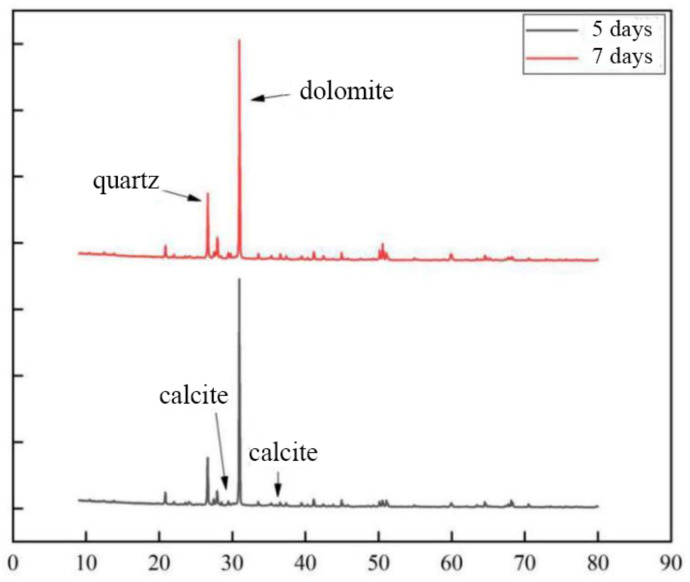
XRD patterns of samples under different curing times.

**Figure 22:**
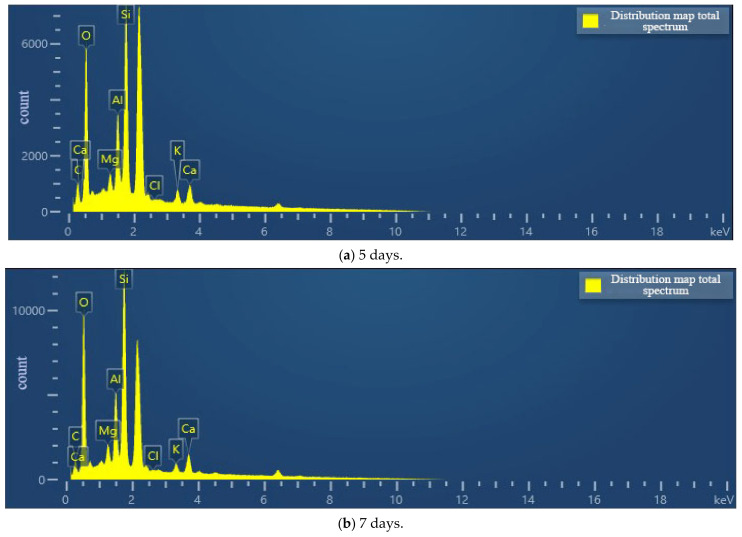
Element distribution diagram for different curing times.

Figure 21 shows that the sample cured for 5 days has more calcium carbonate content than the sample cured for 7 days. Although a lot of calcium ions are precipitated during long-term wet curing, with the increase of wet curing time, the soil is saturated and liquefied. Under the action of water, the calcium carbonate crystals that bind the soil and rock body undergo a reduction reaction, resulting in less calcium carbonate than the calcium carbonate generated after 5 days of curing (Figure 22). Consistent with the mechanical test results in 3.3, it is proved that wet curing for 5 days is the best time for microbial cementation of soil and rock.

## 4. Conclusions

This paper uses the control variable method. By controlling a single variable, unconsolidated and undrained triaxial shear tests were performed on the specimens. The microbial mineralization analysis of a soil–rock mixture and the mechanical properties of the mixture samples were carried out from three aspects of cementation solution pH value, cementation solution concentration, and curing time, Then, the cemented samples were further microscopically studied by SEM and XRD. The main research results are as follows:

i. When the soil–rock mixture is consolidated by micro-organisms, the concentration of the cementation solution should not be too high. Low-concentration cementation solution is beneficial to microbial mineralization, but excessively high concentration inhibits urea hydrolysis and crystallization, the pore filling force between particles is low, and shear deformation is more likely to occur. When 1.25 mol/L bacterial solution and 0.5 mol/L cementation solution were mixed for sample preparation, the resulting sample had the best degree of cementation and the strongest shear resistance.
ii. When the soil–rock mixture is consolidated by micro-organisms, with a pH 6 cementing solution, the bonding effect between the pores is the best, and the deviatoric stress that can be carried is stronger.
iii. When the soil–rock mixture was consolidated by micro-organisms, the soil–rock mixture samples with a short curing time had poorer cementation of microbial mineralization. The micro-organisms did not fully react with the calcium ions in the cementation solution, and the ultimate deviatoric stress was low. With the increase of curing time, the cementing ability of micro-organisms is enhanced, and the bacterial liquid and cementation solution reacts fully to form calcium carbonate crystals and strengthen the consolidation ability of the soil–rock mixed cylinder. If the curing time is too long, the soil is saturated due to being in a humid environment for a long time. Under the action of stress, the pore water between the particles is squeezed without time to discharge, the force between the particles is reduced, and the strength is lost, resulting in the liquefaction of the soil. The overall deviatoric stress is low. When the curing time is 5 days, the bonding and curing ability of the sample is the strongest.
iv. Microscopic results show that microbial mineralization technology fills the pores between particles, and the interaction force between particles is enhanced to achieve the effect of enhancing the strength of the soil–rock mixture.
v. It is feasible to use microbial-induced calcium carbonate precipitation as a technical means to improve the soil–rock mixture. It can be used as an effective measure to improve geotechnical engineering problems and strengthen soil–rock mixture slope to prevent slope instability.

## Figures and Tables

**Table 1:** Basic physical parameters of silt.

Soil Sample Name	Particle Composition mm		Porosity e	Moisture Content W/%	Liquid Limit W_L_/%	Plastic Limit W_P_/%	Plasticity Index IP	Soil Specific Gravity G_S_	Dry Density ρd
Silt	d ≤ 0.075 87%	0.075 < d ≤ 0.1 13%	0.54	5%	21.52	15.54	5.98	2.7	1.7265

**Table 2:** Composition of LB broth liquid medium.

Name	Content
tryptone	10 (L/g)
Yeast Dip Powder	5 (L/g)
calcium chloride	10 (L/g)
final pH	7.0 ± 0.2

**Table 3:** Ultimate deviatoric stress at the failure of soil samples.

Partial Stress (kPa) at Different pH Values				
	**pH 5**	**pH 6**	**pH 7**	**pH 8**
100 kPa	464.6	487.3	445.6	470.8
200 kPa	740.6	806.2	787.9	536
300 kPa	1082.5	1097	926.9	955.2

**Table 4:** Ultimate deviatoric stress at the failure of soil samples.

Partial Stress (kPa) under Different Cementation Concentrations				
	0.25 mol/L	0.5 mol/L	0.75 mol/L	1.0 mol/L
100 kPa	450.9	485.5	409.5	358.4
200 kPa	700.3	739.1	666.9	654
300 kPa	945.4	999.2	956.8	920.7

**Table 5:** Ultimate deviatoric stress at the failure of soil samples.

Partial Stress (kPa) at Different Curing Times				
	1 day	3 days	5 days	7 days
100 kPa	399.8	425.7	470.6	373.5
200 kPa	649.5	745.2	788.4	544.2
300 kPa	771.4	904.2	1321.1	800.6

## Data Availability

All data that support the findings of this study are available from the corresponding author upon reasonable request.
